# The effects of age on sperm quality: an evaluation of 1,500 semen samples

**DOI:** 10.5935/1518-0557.20140002

**Published:** 2014

**Authors:** João Batista A. Oliveira, Claudia G. Petersen, Ana L. Mauri, Laura D. Vagnini, Ricardo L. R. Baruffi, José G. Franco Jr.

**Affiliations:** 1 Center for Human Reproduction Prof. Franco Jr., Ribeirao Preto, SP, Brazil; 2 Paulista Center for Diagnosis Research and Training, Ribeirao Preto, SP, Brazil

**Keywords:** ART, access to ART, Gross Domestic Product, Gini coefficient, Chile

## Abstract

**Objective:**

The aim of this study was to evaluate the effects of aging on semen quality in a population of infertile couples.

**Methods:**

A cross-sectional study of semen samples obtained from 1,500 men randomly selected from couples who attended an infertility clinic was conducted. The analyses were performed using Spearman’s correlation and Mann-Whitney tests. The age groups consisted of men ≤ 35 years, from 36-45 years and > 45 years of age. The semen analysis was performed according to the WHO criteria, and morphology was evaluated using the motile sperm organelle morphology examination (MSOME). The percentages of normal spermatozoa and spermatozoa with large nuclear vacuoles (LNV, occupying > 50% nuclear area) were determined. The percentages of DNA fragmentation were assessed using the TUNEL assay.

**Results:**

A regression analysis revealed that the percentages of LNV spermatozoa and sperm DNA fragmentation positively correlated with age. Conversely, a regression analysis revealed that the percentage of normal sperm, sperm progressive motility and sperm vitality negatively correlated with age. As in the previous test, the analysis by age group showed that there was a significant reduction (*P* < 0.05) in the percentage of normal sperm, sperm progressive motility and sperm vitality as age increased. Conversely, the percentage of spermatozoa with LNVs and sperm DNA fragmentation significantly increased (*P* < 0.05) as age increased.

**Conclusion:**

Semen quality seems to be influenced by aging. The age-related decrease in sperm quality suggests that delaying childbearing, not only for women but also for men, may jeopardize reproductive capacity.

## INTRODUCTION

As a result of the increase in life expectancy and the changing roles of women in society, delaying the onset of reproductive life has become a more common trend, especially in developed countries. However, the increase in the age of couples also brings an increase in reproductive risks. Society has reacted to the increase in maternal age with certain actions such as imposing restrictions on access to fertility treatment for women over the age of 40 years or offering screening tests during pregnancy for fetal malformations. Yet, such precautions are not practiced for older males. Names such as Picasso and Chaplin, who were parents in their old age, are inevitably cited as examples when the reproductive functions of older men are questioned. In addition, statistics show a large number of children born to fathers older than 50 years of age in the general population. However, even considering the discrepancy between the reproductive periods of men and women, the question has been raised as to whether advanced male age is also associated with impaired fertility and/or risks to pregnancy. The evaluation of male fertility is generally based on the examination of sperm parameters. Although there is no known critical age limit for gamete production for men, evidence suggests that there are declines in semen quality (e.g., volume, motility and morphology) and male fertility associated with increasing male age (Andolz *et al*., 1999; Girsh *et al*., 2008; Dain *et al*., 2011; Zhu *et al*., 2011). However, there is no consensus among the published results. Therefore, studies examining the relationship between age and semen quality and/or fertility continue to be important. Conversely, advanced paternal age has been implicated in the increase in the frequencies of abortions (Slama *et al*., 2005; Kleinhaus *et al*., 2006), autosomal dominant diseases, aneuploidy and other diseases (Wyrobek *et al*., 2006; Crosnoe & Kim, 2013; Paul & Robaire, 2013). Advanced male age has also been correlated with infant mortality (Urhoj *et al*., 2014). One plausible explanation for these results is that older men may have more sperm with damaged DNA (Vagnini *et al*., 2007). Chromatin damage has been associated with male infertility, conception problems and problems sustaining pregnancy (Zini & Libman, 2006; Ménézo *et al*., 2007; Das *et al*., 2013). There is also evidence linking DNA damage in sperm with the risk of mutations and birth defects in the offspring (Wyrobek *et al*., 2006; Paul & Robaire, 2013).

Understanding the effects of age on fertility is particularly relevant given that the increase in life expectancy and the availability of assisted reproductive technologies have increased the opportunities for men to have children at older ages. Thus, the aim of this study was to investigate the influence of age on sperm quality in a group of men diagnosed with or under treatment for infertility.

## MATERIAL AND METHODS

### Population

Semen samples (one per subject) were obtained from 1500 men from a random group of couples undergoing infertility investigation and treatment. Written informed consent was obtained from all participants, and the local ethical committee Institutional Review Board approved this study.

### Sample collection

Semen samples were collected in sterile containers by masturbation after a sexual abstinence period of 2-5 days. A portion of each semen sample was used to analyze the following parameters according to the World Health Organization (WHO) guidelines (2010): volume (ml), sperm concentration (x 10^6^/ml), percentage of spermatozoa with progressive motility (rapid + slow progression) and percentage of live spermatozoa (vitality).

The remainder of the semen sample was processed for morphological analysis following motile sperm organelle morphology examination (MSOME) and for sperm DNA fragmentation analysis measured using the TdT (terminal deoxyribonucleotidyl transferase)-mediated dUTP nick-end labeling (TUNEL) assay.

### Determination of morphology by MSOME

The liquefied fresh semen samples were prepared using an Isolate (Irvine Scientific, USA) discontinuous concentration gradient. The final pellet was resuspended in 0.2 ml of modified human tubal fluid (HTF) medium (Irvine Scientific, Santa Ana, CA, USA). An aliquot of 1 µl of sperm cell suspension was transferred to a 5 µl microdroplet of modified HTF medium containing 7% polyvinylpyrrolidone (PVP medium; Irvine Scientific). This microdroplet was placed in a sterile glass dish (Fluorodish; World Precision Instruments, USA) under sterile paraffin oil (Ovoil-100; VitroLife, Goteborg, Sweden). The sperm cells, which were suspended in the microdroplet, were placed on a microscope stage above a U Plan Apochromat 100 x oil/ 1.35 objective lens that had previously been covered by a droplet of immersion oil. With this procedure, the suspended motile sperm cells in the observation droplet could be examined at high magnification using an inverted microscope (Eclipse TE 2000 U; Nikon, Japan) equipped with high-power differential interference contrast optics (DIC/Nomarski). The images were captured by a color video camera that had sufficient resolution to produce high-quality images, which were displayed on a color video monitor. The morphological evaluation was performed on the monitor screen, and the combined calculated magnification was 8450x (total magnification: objective magnification = 100 ×; magnification selector = 1.0 x; video coupler magnification = 1.0 x; calculated video magnification = 84.50).

**Figure 1:**
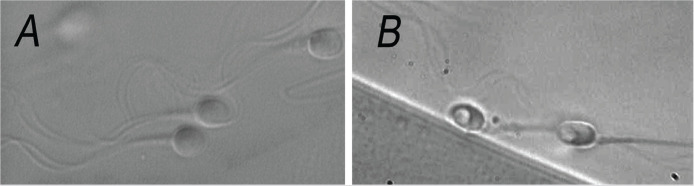
MSOME for human sperm morphology analysis (8,450x). A) Normal spermatozoa observed at high magnification; (B) Spermatozoa with large nuclear vacuoles at high magnification.

Two types of spermatozoa observed via MSOME were counted in this study: normal spermatozoa and spermatozoa with large nuclear vacuoles (LNVs). A spermatozoon was classified as morphologically normal when it exhibited a normal nucleus as well as a normal acrosome, post-acrosomal lamina, neck and tail and had no cytoplasm around the head (Bartoov *et al*., 2002). The morphological state of the nucleus was defined by its shape and chromatin content, as assessed by transmission electron microscopy estimations. The normal nuclear shape was defined as a smooth, symmetric oval. The normal means for length and width were estimated as 4.75 ± 2.8 and 3.28 ± 0.20 µm (Bartoov *et al*., 2002), respectively, and forms classified as abnormal varied by two standard deviations (SD) in at least one of the axes (length: ≥ 5.31 or ≤ 4.19 µm, width: > 3.7 or < 2.9 µm). The criterion for normality of chromatin content was the absence of vacuoles occupying > 4% of the sperm nuclear area. Figure 1A shows normal spermatozoa analyzed by MSOME.

LNV spermatozoa were defined according to the Bartoov modified classification, i.e., the presence of one or more vacuoles occupying > 50% of the sperm nuclear area (visual evaluation aided, if necessary, by a celluloid form of a large vacuole superimposed on the examined cell). Figure 1B shows LNV spermatozoa analyzed using MSOME.

The same technician performed all semen sample evaluations. As in other sperm morphological analyses, each sperm was evaluated/classified individually in MSOME, and the process was carried out directly on the monitor screen. At least 200 motile spermatozoa per sample were evaluated, and the percentages of normal and LNV spermatozoa were determined.

### Determination of DNA damage

DNA fragmentation in the spermatozoa was measured using the TUNEL assay, which was performed using a Cell Death Detection Kit with tetramethylrhodamine-labelled dUTP (Roche, Monza, Italy). TUNEL identifies single- and double-stranded DNA breaks by labeling the free 3’-OH termini with modified nucleotides in an enzymatic reaction with TdT. TdT polymerizes free 3-OH DNA ends in a template-independent manner, incorporating labeled nucleotides. The remaining sperm pellets were smeared on glass slides, air-dried, fixed with 4% paraformaldehyde in PBS at 4°C for 25 min, pH 7.4, and permeabilized with 0.1% Triton X-100 (VETEC Química Fina Ltd, Duque de Caxias, Brazil) in 0.1% sodium citrate at 4°C for 2 min. After washing with PBS, the smears were then processed for the TUNEL assay. The TdT-labeled nucleotide mix was added to each slide and incubated in the dark in a humidified atmosphere for 2 h at 37°C. After stopping the enzyme reaction, the slides were rinsed twice in PBS and then counterstained with Vectashield® Mounting Medium with 4,6-diamidino-2-phenylindole (DAPI; 1.5 µg/ml) (Vector Laboratories, Burlingame, CA, USA). For quantitative evaluation, at least 200 spermatozoa in randomly selected areas on microscope slides were evaluated using a fluorescent microscope, and the percentage of TUNEL-positive spermatozoa was determined. The number of cells per field stained with DAPI (blue) was counted first; the number of cells with red fluorescence (TUNEL positive) was expressed as a percentage of the total sample. Controls were included in every experiment: for the negative control, TdT was omitted from the nucleotide mix. Positive controls were generated by pre-incubating the fixed and permeabilized sperm cells using DNaseI at 1 mg/ml (New England Biolabs, Inc, Ipswich, MA, USA) for 30 min at 37°C. The TUNEL labeling of positive controls varied from 89-98% of the cells. The same technician, blinded to the subject identity, performed all the examinations.

### Statistical analysis

The data were analyzed using the StatsDirect statistical software (Cheshire, UK). The Mann-Whitney U test, Student’s t-test and the chi-squared test were used as appropriate. Correlations were performed using the Spearman rank correlation test. Patient age and percentages of normal and LNV spermatozoa, volume, sperm concentration, percentage of progressive motility and vitality were treated as continuous variables for the regression and correlation analysis. For two-group comparisons, the following ages were used as cut-off points to divide the subjects into groups: Group I: ≤ 35 years, Group II: 36-45 years, and Group III: > 45 years. The level of significance was set at *P* < 0.05.

## RESULTS

Table 1 summarizes the general characteristics of the study population. The comparison between the three age groups showed that a significantly higher proportion of older men had fathered at least one child (or generated a pregnancy that had ended in miscarriage), spontaneously or after fertility treatment, compared with the younger men. Similarly, an increase in the length of the infertile period was also observed with increasing age.

**Table 1:** General characteristics of the three age groups studied

Characteristic	Age Group				
	Total	≤ 35 years	36-45 years	> 45 years	P
Patients (n)	1500	597	727	176	
Age (years)	37.7 ± 6.7 (22-76)	31.8 ± 2.7 (22-35)	39.5 ± 2.7 (36-45)	50.6 ± 5.3 (46-76)	
Body mass index	28.3 ± 4.3 (17-44.1)	28.6 ± 4.4 (17-43.1)	28.2 ± 4.1 (18.9-42.9)	28.2 ± 4.4 (19.8-44.1)	ns
Fathered at least one child	33.0% (495/1500)	21.8%^A,B^ (130/597)	37.3%^A,C^ (271/727)	53.4%^B,C^ (94/176)	^A,B^< 0.0001 ^C^0.0001
Duration of infertility (years)	3.9 ± 3.0 (1-11)	3.1 ± 2.0^A,B^ (1-8)	4.1 ± 3.0^A^ (2-10)	5.4 ± 4.5^B^ (1-11)	^A,B^< 0.0001
Varicocele	17.1% (257/1500)	15.9% (95/597)	18.2% (132/727)	17% (30/176)	ns
Tobacco use	11.7% (176/1500)	13.4% (80/597)	10.5% (76/727)	11.4% (20/176)	ns
Regular alcohol use	64.1% (962/1500)	64.5% (385/597)	64.8% (471/727)	60.2% (106/176)	ns
Vitamin supplement use	15.1% (227/1500)	15.2% (91/597)	14.7% (107/727)	16.5% (29/176)	ns

Values within rows with the same superscript letter were significantly different ns: Not significant

Table 2 summarizes the results of the two-group comparisons. An influence of aging on sperm concentration was not observed. However, the semen volume, the progressive motility and the vitality of sperm worsened with age. The overall percentage of sperm volume was 2.9 ± 1.4 ml (range 0.5-9.5 ml). The mean volume was 3.0 ± 1.4 ml (range 0.5-9.5 ml) in Group I, 2.9 ± 1.3 ml (range 0.5-8.5ml) in Group II and 2.5 ± 1.4 ml (range 0.5-7 ml) in Group III. There was no difference in the semen volume between the younger (I and II) groups (*P* = 0.56). The volume in the older group (III) was significantly lower than those in both of the younger (I and II) groups (P < 0.0001 and *P* < 0.0001, respectively). The overall percentage of sperm progressive motility was 56.9 ± 17.3% (range 0-95%). The mean progressive motility was 58.9 ± 16.5% (range 0-95%) in Group I, 56.3 ± 17.2% (range 0-95%) in Group II and 51.8 ± 19.5% (range 0-85%) in Group III.

**Table 2:** Analysis of the three age groups studied

Characteristic	Age Group				
	Total	≤ 35 years	36-45 years	> 45 years	P
Patients (*n*)	1500	597	727	176	
Abstinence (days)	3.6 ± 1.0 (2-5)	3.2 ± 1.0 (2-5)	3.5 ± 1.0 (2-5)	4.0 ± 2.0 (2-5)	ns
Volume* (ml)	2.9 ± 1.4 (0.5-9.5)	3.0 ± 1.4^A^ (0.5-9.5)	2.9 ± 1.3^B^ (0.5-8.5)	2.5 ± 1.4^A,B^ (0.5-7)	^A,B^< 0.0001
Concentration* (x 10^6^/ml)	62.5 ± 50.8 (0.1-390)	60.6 ± 49.4 (0.1-305)	64.1 ± 52.1 (0.1-390)	61.2 ± 49.8 (0.1-280)	ns
Motility* (rapid + slow progression) %	56.9 ± 17.3 (0-95)	58.9 ± 16.5^A,B^ (0-95)	56.3 ± 17.2^A,C^ (0-95)	51.8 ± 19.5^B,C^ (0-85)	^A^< 0.003 ^B^< 0.0001^C^0.01
Vitality* (%)	64.6 ± 15.6 (0-98)	66.7 ± 14.6^A,B^ (0-97)	64.0 ± 15.3^A,C^ (0-98)	60.1 ± 18.3^B,C^ (0-86)	^A^0.0002 ^B^< 0.0001^C^0.03
Leukocytes (x 10^6^)	0.4 ± 2.2	0.5 ± 3.4	0.4 ± 0.9	0.3 ± 0.5	ns
Sperm morphology** (%) Normal spermatozoa Spermatozoa with LNV	1.03 ± 1.7 (0-15) 31.6 ± 19.7 (2-100)	1.11 ± 1.9^A^ (0-15) 30.4 ± 18.7^A^ (3-98)	1.05 ± 1.7 (0-11) 31.4 ± 19.5^B^ (2-100)	0.69 ± 1.0^A^ (0-8) 36.5 ± 23.1^A,B^ (5-100)	^A^0.03 ^A^0.001 ^B^0.0050
Sperm DNA fragmentation (%)	16.2 ± 9.3 (3-60)	15.3 ± 9.0^A,B^ (3-59)	16.7 ± 9.7^A^ (3-60)	16.7 ± 8.5^B^ (4-41)	^A^0.008 ^B^0.02

* Categorized according to the World Health Organization guidelines

** MSOME criteria

Values within rows with the same superscript letter were significantly different ns: not significant

There were significant differences in the progressive motility among the three groups (Group I X Group II: *P* = 0.003; Group I X Group III: *P* = < 0.0001; Group II X Group III: *P* = 0.01). The overall percentage of sperm vitality was 64.6 ± 15.6% (range 0-98%). The mean vitality was 66.7 ± 14.6% (range 0-97%) in Group I, 64.0 ± 15.3% (range 0-98%) in Group II and 60.1 ± 18.3% (range 0-86%) in Group III. Similar to sperm motility, there were significant differences in the sperm vitality among the three groups (Group I X Group II *P* = 0.0002; Group I X Group III *P* = < 0.0001; Group II X Group III *P* = 0.03).

The overall percentage of sperm with normal form, as analyzed by MSOME, was 1.03 ± 1.7% (range 0-15%). The mean percentage of sperm with a normal form was 1.11 ± 1.9% (range 0-15%) in Group I, 1.05 ± 1.7% (range 0-11%) in Group II and 0.69 ± 1.0 (range 0-8%) in Group III. There was no difference in the percentage of normal sperm in the two younger (I and II) groups (*P* = 0.49) or between groups II and III (*P* = 0.10). However, the percentage of normal sperm in the older group (III) was significantly lower than those in either of the younger (I) groups (*P* = 0.03). The overall percentage of LNV spermatozoa was 31.6 ± 19.7% (range 2-100%). The mean percentages of LNV spermatozoa were 30.4 ± 18.7% (range 3-98%) in Group I, 31.4 ± 19.5% (range 2-100%) in Group II and 36.5 ± 23.1% (range 5-100%) in Group III. There was no difference in the percentage of spermatozoa with LNVs between the younger (I and II) groups (*P* = 0.47). However, the percentage of spermatozoa with LNVs in the older group (III) was significantly higher than those in both of the younger (I and II) groups (*P* = 0.001 and *P* = 0.005, respectively).

The overall percentage of sperm DNA fragmentation was 16.2 ± 9.3% (range 3-60%). The mean DNA fragmentation was 15.3 ± 9.0% (range 3-59%) in Group I, 16.7 ± 9.7% (range 3-60%) in Group II and 16.7 ± 8.5% (range 4-41%) in Group III. The percentage of sperm DNA fragmentation was significantly lower in Group I than Group II (*P* = 0.008) or III (*P* = 0.02). However, there was no difference in DNA fragmentation between Groups II and III (*P* = 0.64).

**Figure 2:**
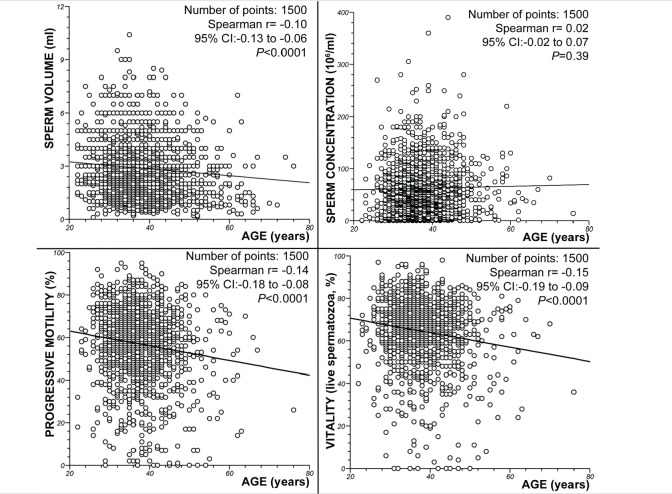
Spearman rank correlation test. Correlations between age and pH, volume (ml), sperm concentration (x 10^6^/ml), percentage of spermatozoa with progressive motility (rapid + slow progression), number of leukocytes (x 10^6^) and percentage of live spermatozoa (vitality).

The regression analysis did not show a correlation between age and sperm concentration (*P* = 0.39; Spearman’s rank correlation r = 0.02). However, significant decreases in the semen volume (*P* < 0.0001; Spearman’s rank correlation r =-0.10), progressive motility (*P* < 0.0001; Spearman’s rank correlation r =-0.14) and sperm vitality (*P* < 0.0001; Spearman’s rank correlation r=-0.15) with increasing male age were observed. Figure 2 summarizes these results.

**Figure 3:**
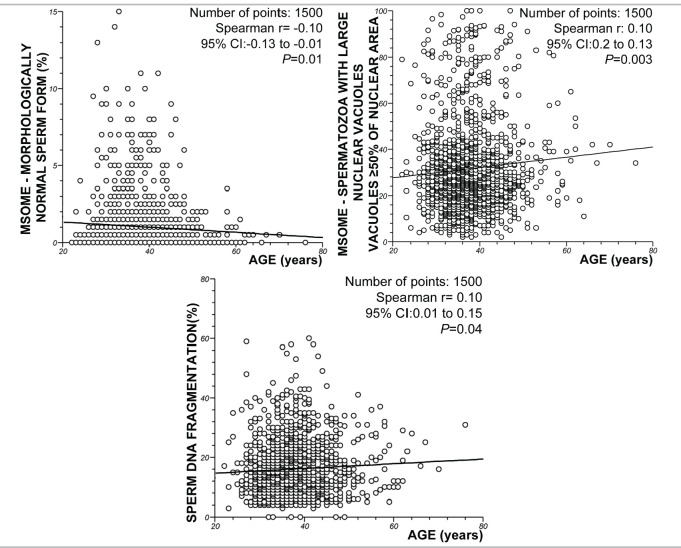
Spearman rank correlation test. Correlations between age and sperm morphology and sperm DNA fragmentation.

In relation to sperm morphology, the regression analysis demonstrated a significant decrease in the incidence of normally formed sperm with increasing male age (*P* = 0.01; Spearman’s rank correlation r =-0.10). However, there was a significant positive correlation between the percentage of spermatozoa with LNVs and male age (*P* = 0.003, Spearman rank correlation r = 0.10). Similarly, the regression analysis also demonstrated a significant increase in sperm DNA fragmentation with age (*P* = 0.02; Spearman’s rank correlation coefficient, r = 0.10). Figure 3 summarizes these results.

## DISCUSSION

The effect of paternal age on semen quality has been discussed in the literature, but the results are not consistent. Our results found that increased age is associated with decreases in semen volume, sperm vitality and sperm progressive motility. Several studies have reported similar results, noting an inverse correlation between semen volume (Spandorfer *et al*., 1998; Andolz *et al*., 1999; Moskovtsev *et al*., 2009; Brahem *et al*., 2011; Dain *et al*., 2011; Stone *et al*., 2013), motility (Moskovtsev *et al*., 2009; Winkle *et al*., 2009; Zhu *et al*., 2011; Stone *et al*., 2013) and sperm vitality (Moskovtsev *et al*., 2009; Brahem *et al*., 2011; Zhu *et al*., 2011; Stone *et al*., 2013) with male age. There is no consensus in the literature, as some authors have not observed any correlation between age and some of these semen parameters (Berling & Wölner-Hanssen, 1997; Spandorfer *et al*., 1998; Frattarelli *et al*., 2008; Nijs *et al*., 2011; Fréour *et al*., 2012). Conversely, in this study, the sperm concentration did not show a significant variation with age, in agreement with previous studies that showed little or no association between age and sperm concentration (Spandorfer *et al*., 1998; Bellver *et al*., 2008; Frattarelli *et al*., 2008; Dain *et al*., 2011; Nijs *et al*., 2011; Fréour *et al*., 2012). There is again no consensus in the literature. Some studies have reported a decrease in the concentration of spermatozoa with increasing age (Luna *et al*., 2009; Stone *et al*., 2013), whereas others have observed an increase in sperm concentration (Andolz *et al*., 1999; Brahem *et al*., 2011). It should be noted, however, that the design of the different published studies and their respective populations are quite heterogeneous. Whereas some used volunteers, others based their research on populations under treatment in infertility clinics. This difference complicates the interpretation of these results.

The pathophysiology of the impact of age on semen parameters can occur due to both the specific effects of age alone and associated factors such as obesity, infections of the reproductive glands or the accumulation of toxic substances. It is important to note that a significant number of these studies did not control for potential confounding factors. In our study, factors such as tobacco and alcohol use, the presence of varicocele or the intake of vitamins did not appear to influence the results. Similarly, no differences were observed in the numbers of leukocytes in the semen.

Conversely, a longer duration of abstinence may be responsible for the differences in the results. The length of sexual abstinence was controlled (2-5 days) so that it would not bias the results. Further comparisons were made between the semen parameters to better evaluate the semen parameters and the different numbers of days of abstinence (2, 3, 4 and 5). No significant differences were observed. Innovative methods for selecting spermatozoa in assisted reproductive techniques (ARTs) have been published, providing new insights into the correlations between sperm quality and clinical outcomes. To test the hypothesis that the subtle defects in semen organelles are associated with the ART outcomes, Bartoov *et al*., (2002) proposed a novel method to morphologically evaluate sperm in real time and at high magnification (> 6,000 x) called motile sperm organelle morphology examination (MSOME). In MSOME, the most important predictor of sperm quality is the extent of the impairment of the sperm head due to the presence of vacuoles. Vacuoles, which are best observed at high magnification, appear to correlate with abnormal chromatin packaging or the denaturation and fragmentation of sperm DNA (Franco *et al*., 2008, 2012; Boitrelle *et al*., 2013).

Regarding the morphology of the spermatozoa, our results show a significant decrease (*P* = 0.01) in the percentage of morphologically normal spermatozoa and a significant increase (*P* = 0.003) in the percentage of LNV spermatozoa with increasing age. These results confirm the previous evaluation by our group (Silva *et al*., 2012), which also showed a significant correlation between age and the percentages of normal spermatozoa and spermatozoa with LNVs (r =-1.0, *P* = 0.0015 and r = 0:1A0, *P* = 0.0012, respectively). Similar to the results of this study, Braga *et al*. (2011) analyzed the relationship between spermatozoa morphology evaluated using MSOME and age and reported a positive correlation between age and the presence of nuclear vacuoles (large vacuoles *P* < 0.001; small vacuoles *P* < 0.001). However, these authors reported that there was no correlation between the frequency of morphologically normal spermatozoa, as defined by MSOME, and male age (*P* = 0.715). Importantly, these authors defined the MSOME criteria for morphological normality of the spermatozoa nuclei according to Cassuto *et al*., (2009), whereas we used the criteria proposed by Bartoov *et al*. (2002). This difference may explain the conflicting results. Unfortunately, MSOME is not normally used other than in the selection of spermatozoa for ART. Indeed, to our knowledge, only the cited studies examined the relationship between sperm morphology evaluated using MSOME and male age. Our results contrast with those of several studies that reported no relationship between age and sperm morphology (Kidd *et al*., 2001; Brahem *et al*., 2011; Dain *et al*., 2011; Nijs *et al*., 2011). But, variations in the criteria used to analyze the sperm morphology in each of these studies may explain this discrepancy (Kidd *et al*., 2001), especially considering that the count of specific abnormalities can vary depending on the classification used. Conversely, MSOME gives particular importance to the nuclear morphology of sperm. Our data are consistent with those of several other studies that used other semen morphological evaluation criteria besides MSOME (Andolz *et al*., 1999; Girsh *et al*., 2008; Zhu *et al*., 2011; Stone *et al*., 2013).

The present study also observed an increase in the fragmentation of sperm DNA associated with an increase in patient age. However, the impact of age on the fragmentation of sperm DNA still remains the object of study. Several techniques are currently available to evaluate damage to sperm DNA. Using the TUNEL assay, we found a significant positive association between paternal age and the levels of DNA fragmentation.

The correlation between age and sperm DNA fragmentation using the TUNEL assay was previously observed in a prior study by our group (Vagnini *et al*., 2007). Our results are not consistent with the data reported by Sun *et al*. (1997), Colin *et al*. (2010) and Brahem *et al*. (2011); they used the TUNEL assay and did not observe a significant relationship between age and DNA damage. Besides their sample sizes were much smaller (Sun *et al*., 1997; Colin *et al*., 2010; Brahem *et al*., 2011) than the sample size in the present study, DNA damage was evaluated after washing the sperm according to the swim-up method (Sun *et al*., 1997), a procedure that can significantly decrease the proportion of spermatozoa with DNA fragmentation. Furthermore, our results are consistent with the data reported by Plastira *et al*. (2007) and Varshini *et al*. (2012) who used the TUNEL assay and reported that male aging affects the integrity of spermatozoa chromatin in the infertile population. Likewise, other researchers (Singh *et al*., 2003; Sati *et al*., 2008; Moskovtsev *et al*., 2009) have reported results similar to our findings using different assays to measure sperm DNA damage in infertile and non-infertile populations. However other authors having reported conflicting results. Nijs *et al*. (2011) using the sperm chromatin structure assay (SCSA), reported no significant male age-related increase in the DNA fragmentation index (DFI). Moreover, Winkle *et al*. (2009), using propidium iodide staining and flow cytometry, reported that male age does not affect the number of spermatozoa with fragmented DNA. We should consider that the majority of studies show a direct correlation between male age and the damage of sperm DNA.

Variations in the results in the literature may be related to the technique used and/or the population analyzed.

Despite the majority of the correlations between age and sperm parameters being significant (< 0.05) in our study, they could be considered weak (Spearman’s r <0.4). However, the correlations were quite similar to those found by different authors. Brahem *et al*., (2011) reported values of r = -0.183 (*P* = 0.032), r = -0.219 (*P* = 0.001) and r = 0.196 (*P* = 0.021) for the correlations between age and semen volume, vitality and sperm concentration, respectively. The relationship between sperm morphology and age was similar to those found by authors using different sperm classification criteria: Brahem *et al*. (2011) r = 0.026, not significant; Andolz *et al*. (1999) r^2^ = 0.020, *P* < 0.001; and Braga *et al*. (2011) r^2^ = 0.118, *P* < 0.001. Similarly, for DNA fragmentation, other authors found results similar to ours: Sun *et al*. (1997), r = 0.06, not significant; Moskovtsev *et al*. (2009), r = 0.24, significant. On the other hand others have reported stronger correlations between age and DNA fragmentation: Singh *et al*. (2003), r = 0.56, *P* < 0.001; and Wyrobek *et al*. (2006), r = 0.64- 0.72, *P* < 0.001. It is likely that other factors influence the correlations between age and sperm parameters. Furthermore, differences in sample size, evaluation methods, and statistical analysis methods likely contribute to the differences between these studies. Unfortunately, not all studies have used this type of statistical analysis, which makes the interpretation of these correlation values challenging.

In conclusion, semen quality seems to be influenced by aging. The age-related decrease in sperm quality suggests that delaying childbearing, not only for women but also for men, may jeopardize reproductive capacity.

Considering the relationship with DNA damage, these age-related changes suggest that advanced paternal age may be associated with an increased risk of unsuccessful and abnormal pregnancy as a consequence of fertilization with damaged spermatozoa. This information may be useful in the clinical management of male infertility.
